# Dr. Martin Bessemer

**Published:** 1917-01

**Authors:** 


### OBITUARY

Readers are requested to report promptly the death of all physicians in Western New York, or former residents of this region, or graduates of any medical school in Western New York, and to notify the families of the deceased of our desire to publish adequate Obituary notices.

---

**Dr. Martin Bessemer,** Cleveland Homoeopathic 1875, died at his home in Ithaca Dec. 3, aged 66. He was born in the nearby town of Dryden, and studied at Eastman Academy of Poughkeepsie before taking his actual medical course. Death was due to spinal injury received in a fall at Pulaski last spring.

